# Mapping pathogen genomics training provision: a structured analysis within a global consortium network

**DOI:** 10.3389/fpubh.2026.1768827

**Published:** 2026-04-29

**Authors:** Alice Matimba, Kirsty Lee Garson, Nídia S. Trovão, Siddiqah George, Yiqun Tony Li, Daniel Evans, Jorge Batista da Rocha, James Otieno, Jolynne Mokaya, Tracey Calvert-Joshua, Heather M. Blankenship, Nicola Mulder

**Affiliations:** 1 Wellcome Sanger Institute, Hinxton, United Kingdom; 2 Computational Biology Division, Department of Integrative Biomedical Sciences, University of Cape Town, Cape Town, South Africa; 3 Fogarty International Center, National Institutes of Health, Bethesda, MD, United States; 4 Minnesota Department of Health, Saint Paul, MN, United States; 5 Epidemic and Pandemic Management Department, World Health Organization, Geneva, Switzerland; 6 PHA4GE, Cape Town, South Africa; 7 Michigan Department of Health and Human Services, Lansing, MI, United States

**Keywords:** bioinformatics training, genomic surveillance, pathogen genomics, public health workforce development, training programs

## Abstract

**Background:**

Pathogen genomics plays a central role in infectious disease surveillance and outbreak response. However, information about available training initiatives remains fragmented, limiting visibility into how programmes are structured, delivered, and assessed.

**Methods:**

We conducted a structured survey to characterise pathogen genomics training initiatives identified through the PHA4GE Training and Workforce Development Working Group and affiliated professional networks.

**Results:**

Eighty-one courses were analysed representing pathogen genomics training initiatives from 17 countries. Over half (52%) targeted academic or research audiences and 46% targeted public health professionals. Majority of courses were delivered as short, limited-duration standalone courses. Beginner-level courses accounted for 58% of offerings, whereas only 6% were classified as advanced. Bioinformatics or genomic data analysis was widely represented (72%), while specialised areas such as biostatistics and systems administration were less frequently included. Nearly half (48%) of courses focused on broadly applicable genomic methods rather than being restricted to a single pathogen. Among courses centred on specific organisms, viral pathogen themes were most commonly represented. Over one-third of courses (38%) did not include structured assessments, with only 7% incorporating quizzes or exams. Most courses relied on local computing resources such as laptops or desktops during delivery (93%). Use of high-performance computing (HPC) and cloud platforms was limited during training but was higher after training, with 37% and 39% of courses indicating use, respectively.

**Conclusion:**

This landscape analysis identifies structural patterns, including geographic concentration of providers, predominance of introductory formats, variability in assessment practices and in the use of advanced computing infrastructure across training phases. The findings provide empirical insight into characteristics of pathogen genomics training that may inform efforts to strengthen coordinated and sustainable workforce development strategies.

## Introduction

Pathogen genomics now plays a central role in infectious disease surveillance and outbreak response, informing real-time investigations, strengthening surveillance systems, and supporting clinical and public health decision-making (1–4). In this article, pathogen genomics is defined as the application of genomic technologies and bioinformatics to sequence and analyse genetic material of pathogens, including viruses, bacteria, fungi, protozoan parasites, and helminths (1, 2, 5). This encompasses wet-lab processes (sample processing, library preparation, and sequencing), bioinformatics analysis (data processing, genome assembly, variant calling, and phylogenetic analysis), data interpretation and translating genomic data into actionable insights for public health, clinical, and research contexts.

As demand for genomics-driven evidence grows, so does the need for effective, accessible, and scalable training programmes that build the capacity of public health professionals, researchers, and laboratory scientists worldwide (4, 6). The COVID-19 pandemic accelerated investment in pathogen genomics training and catalysed a shift from traditional in-person delivery toward more flexible digital and hybrid learning models (3, 7, 8). In some contexts, this transition contributed to strengthened local bioinformatics capacity and expanded engagement with genomic technologies (4, 9, 10).

Despite these advances, disparities in access to genomics expertise and workforce development persist (3–5, 8, 11). Many established training initiatives have historically originated from Europe and North America, where sustained funding, infrastructure and institutional networks have supported programme development. However, locally and regionally led genomics programmes are increasingly emerging across Africa, Asia, and Latin America reflecting expanding infrastructure, growing technical expertise, and strengthening collaborative networks (4, 8, 11, 12). This evolving landscape reflects increasing regional leadership in genomics workforce development.

Pathogen genomics training is delivered by a diverse range of organizations, including universities, research institutes, public health agencies, non-governmental organizations, and international networks operating at institutional, national, regional, and transnational levels (3–5, 8, 11, 13, 14). However, information about these programmes is often dispersed across institutional websites and within professional communities, limiting their visibility. This fragmentation makes it difficult for learners to identify appropriate opportunities, for educators to build on shared resources, and for funders and policy-makers to understand the characteristics of the broader training landscape.

While pathogen genomics training initiatives exist across diverse contexts, there has been limited systematic mapping of how these programmes are distributed, delivered, and structured. To begin addressing this gap, the Training & Workforce Development (TWD) Working Group of the Public Health Alliance for Genomic Epidemiology (PHA4GE) Consortium conducted a survey-based analysis of pathogen genomics training programmes identified through consortium members and affiliated professional networks. The study examined key features of these programmes and provides a structured snapshot of training provision within this network, highlighting patterns that may inform coordination and workforce development efforts.

## Methods

### Survey instruments

Two structured surveys were developed and implemented by the PHA4GE Training and Workforce Development (TWD) Working Group. The surveys were designed to collect detailed information on (i) training course characteristics and (ii) the computational environments used to support training delivery. Both surveys were administered in English.

The first survey (Supplementary File S1) collected information on pathogen genomics training courses, including

- Course details: Title, institution type, and intended audience
- Focus areas: Topics covered by the course, including genome sequencing, epidemiology, bioinformatics, and specialised areas such as biostatistics and systems administration.
- Course structure: Course level (beginner, intermediate, advanced), delivery mode (face-to-face, live remote, pre-recorded), and type of enrolment (open access, application required, selective admission).
- Assessment and accreditation: Presence of formal assessments, type of certification or accreditation offered, and frequency of course delivery (e.g., ongoing, scheduled intervals).
- Accessibility features such as availability of multilingual support (e.g., subtitles or transcripts), geographic relevance, and whether course materials were reusable or open-access.

A second survey (Supplementary File S2) was distributed to respondents of the first survey to gather information on the infrastructure and technical requirements associated with course delivery. This survey captured infrastructure use before, during, and after training, including local computing resources, on-premise high-performance computing (HPC), and public or private cloud platforms.

### Survey respondents and dissemination

The primary target group comprised PHA4GE Consortium members and affiliated professional networks. The survey invitation was circulated via email to TWD Working Group members and other individuals engaged in genomics training, research, and public health workforce development. At the time of survey dissemination, TWD Working Group included representation from 33 countries across six regions, including 17 countries in Africa, 7 in Asia, 6 in Europe, 2 in North America, 1 in Oceania, and 1 in Latin America, reflecting broad geographic distribution within the network. Respondents were asked to complete a separate survey for each training course they had delivered to ensure course-level specificity and minimise duplication. Since dissemination relied on professional and consortium networks, the data collected reflects training initiatives identified within this sampling frame.

### Data collection and management

Both surveys were administered online using the REDCap (Research Electronic Data Capture) data management platform (15). Data were collected between March 2021 and September 2023. All responses were self-reported by training organisers and stored securely in accordance with institutional data governance policies.

### Data analysis

Survey responses (Supplementary File S3) were analysed descriptively to identify patterns in geographic distribution, course structure, topic coverage, assessment practices, and infrastructure use within the surveyed training initiatives. Percentages were calculated using the relevant denominators (e.g., total courses or infrastructure subset, where applicable). The findings were synthesised to describe structural characteristics of training provision within the defined sampling frame.

### Data dissemination

To improve discoverability of pathogen genomics training initiatives, the course information collected through this study was curated onto the NGS Academy platform (https://uct-cbio.github.io/ngs-academy/courses/other-courses). This searchable repository provides access to information on training courses captured through the survey.

## Results

### Geographic distribution of survey responses

**Figure 1:**
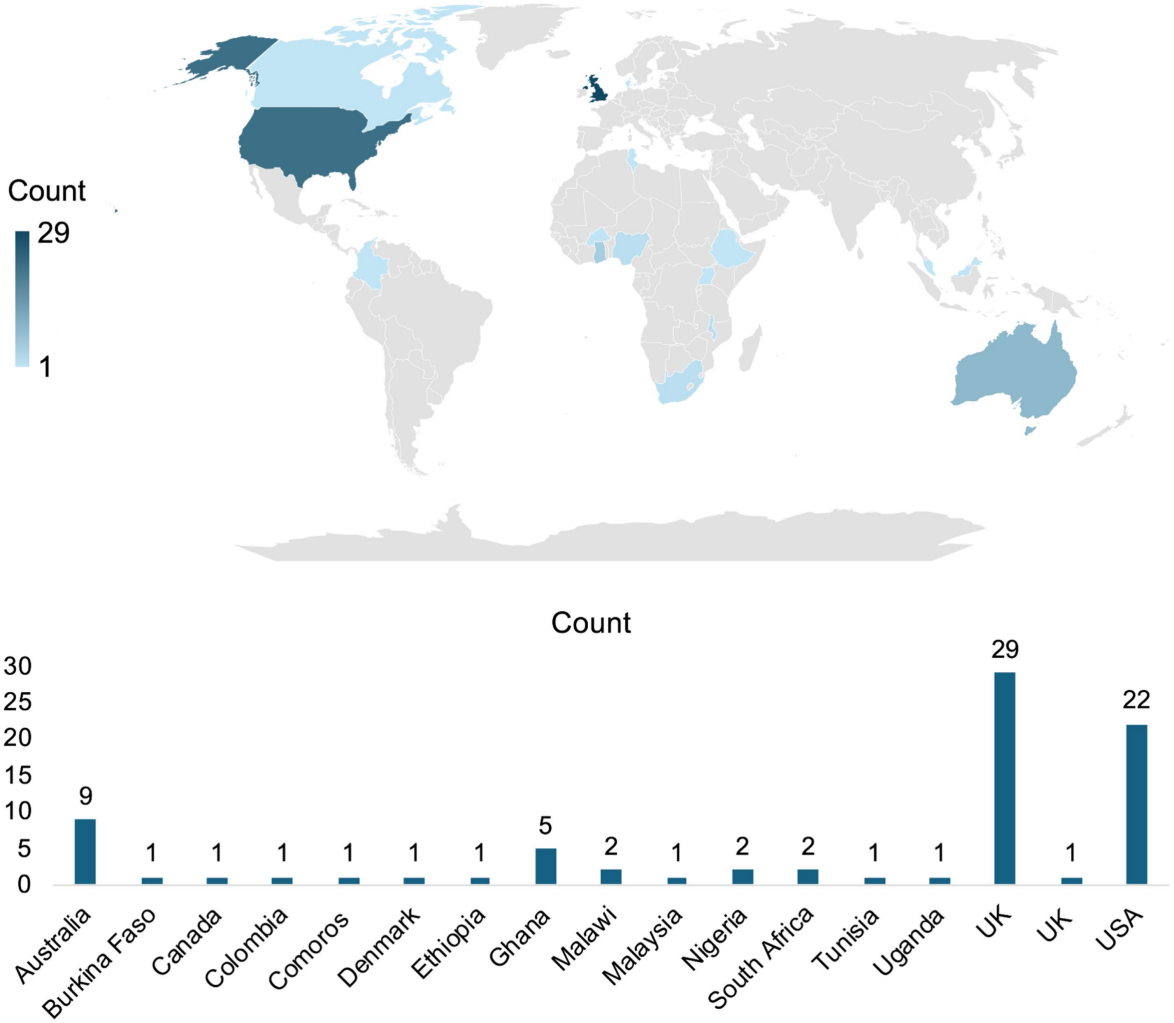
Geographic distribution of training provider locations represented in the surveyed dataset. The map shows the institutional base of training providers associated with the 81 surveyed courses across 17 countries, identified through survey dissemination via the PHA4GE Training & Workforce Development Working Group and affiliated networks (March 2021–September 2023). Countries are shaded according to the number of courses reported by providers based in each location. This figure reflects the location of the training provider and not necessarily the physical location of course delivery. The accompanying bar chart presents the corresponding course counts per provider country.

Survey responses corresponded to 88 courses, of which 7 were excluded due to incomplete information or duplication, resulting in a final dataset of 81 unique courses (Supplementary File S3). These courses were delivered by 28 training provider institutions located across 17 countries (Figure 1). Institutions were based in 8 African countries, 2 each in Europe, Asia, North America, and South America, and 1 in Oceania. This analysis mapped the institutional base of training provision or organization, rather than the geographic location of learners who were recipients. Training providers located in the United Kingdom (*n* = 29) and the United States (*n* = 22) were disproportionately represented, together accounting for 63% of the courses identified. This geographic skew reflects the distribution of training providers captured within the PHA4GE, which should be considered when interpreting regional patterns in relation to broader global training activity or participant distribution. The institutional base of the training provider does not necessarily reflect the geographic distribution of course participants or learners.

### Course format and delivery approaches

Face-to-face (in-person) delivery was reported in 38 courses (47%). Live remote (synchronous online) delivery was reported in 35 courses (43%), and pre-recorded (asynchronous digital content) components in 31 courses (38%). These three primary modalities were frequently combined, with 33 courses (41%) incorporating more than one format. Twelve courses (15%) were delivered exclusively as standalone MOOCs, and 10 courses (12%) reported bespoke, learner-responsive delivery models.

Majority of the face-to-face format courses were delivered within the country where the institution is located, including courses hosted in the United States, United Kingdom, South Africa, Malaysia, Canada, Burkina Faso, and Australia. However, 20 courses were delivered outside the provider’s home country or across multiple geographic regions. Examples of delivery outside the provider’s home country included courses delivered from the United States to Ethiopia and the United Arab Emirates, and from the United Kingdom to sites in Africa. Several courses were delivered in-person across more than one regional location over different delivery cycles, including Africa, Asia, Latin America. In addition, nine courses from a single provider reported delivery locations that varied according to participant demand, rather than fixed geographic sites or delivery mode. Information on the geographic location of learners participating in online or live remote courses was not collected; therefore, the distribution of participants in non–face-to-face formats cannot be inferred from these data.

### Course organization and instructional structure

Scheduling patterns varied across programmes. Thirty-seven courses (46%) were presented at regular intervals (e.g., annual or biennial offerings). Eleven courses (14%) were constantly available, and nine courses (11%) made materials freely accessible online independent of scheduled delivery. Nineteen courses (23%) were conditionally or demand-driven, including those delivered upon request, dependent on funding availability, or offered according to trainee demand. No information was provided for the rest of the courses.

Six courses (7.4%) were delivered over hours, typically as short seminars or modular online units requiring fewer than 20 h of total engagement. Thirty-two courses (39.5%) were structured as short in-person or virtual workshops lasting several days, most commonly between 3 and 7 days. Thirty-seven courses (45.7%) extended over one or more weeks. Within this group, durations ranged from one to two-week intensive workshops to structured multi-week programmes lasting three to eight weeks, and in some cases up to twelve weeks. Four courses (4.9%) represented longer-term initiatives extending over several months. Two courses (2.5%) did not provide sufficient information to categorise duration.

Instructional pacing also differed across programmes. Fifty-eight courses (72%) combined self-paced components with instructor-led sessions. Thirteen courses (16%) were exclusively instructor-led, while 10 courses (12%) were fully self-paced.

### Accessibility and sustainability of course materials

Reusability of course materials varied. Forty-seven courses (58%) permitted reuse by trainees or instructors. Twenty-one courses (26%) restricted reuse, 12 courses (15%) allowed limited or conditional reuse (e.g., requiring attribution or institutional permission), and one course (1%) did not specify reuse conditions.

Reporting on multilingual accessibility and support was heterogeneous and varied across programmes. Twenty-seven courses (33%) reported providing subtitles, transcripts, translated materials, or interpretation support in one or more additional languages. Twenty-three courses (28%) explicitly indicated no additional language support beyond the primary language of instruction, often specifying English-only delivery. A further 10 courses (12%) specified English as the language of instruction without clarifying whether additional accessibility features were available. For 21 courses, responses were insufficiently detailed to categorise language support definitively.

### Training contexts and target audiences

The survey captured a range of pathogen genomics training programmes delivered across research and public health contexts. Forty-two courses (52%) were primarily designed for individuals based in academic or research institutions, including bioinformaticians, experimental biologists, and graduate to postdoctoral-level scientists. These courses emphasised development of technical and analytical competencies relevant to research environments.

Thirty-seven courses (46%) were primarily designed for healthcare and public health professionals, reflecting the increasing integration of genomics into disease surveillance and outbreak response activities. Notably, courses classified as research-focused were often described as applicable to public health settings, indicating overlap in training requirements across sectors. Two courses provided general theoretical instruction without reference to a specific sector.

### Course content and proficiency levels

**Figure 2:**
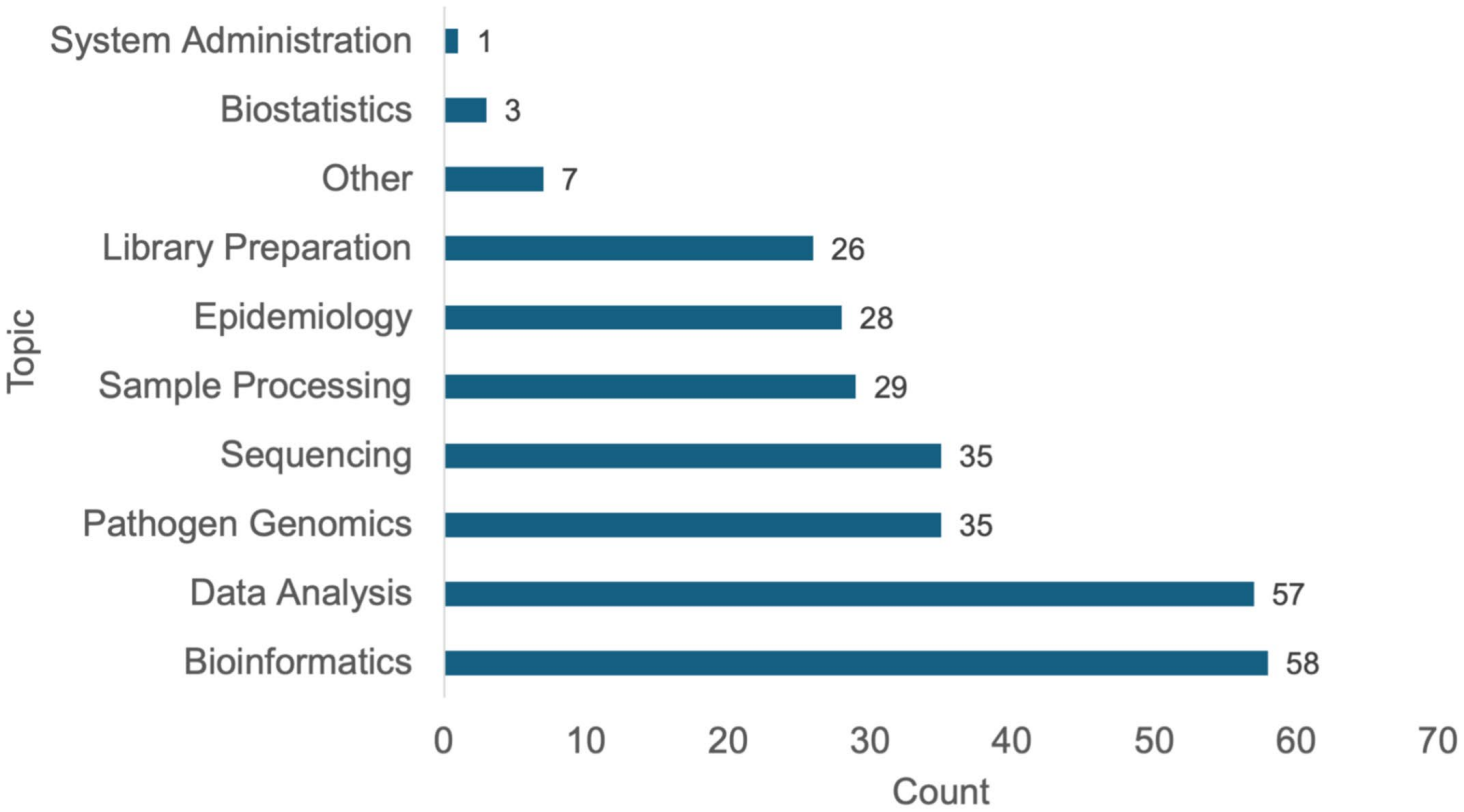
Distribution of topics covered in pathogen genomics training courses. The bar chart displays the number of surveyed courses (*n* = 81) reporting inclusion of specific topic areas in their curriculum.

A range of topics were covered across the surveyed courses (Figure 2). Bioinformatics and data analysis were the most frequently represented areas, included in 58 courses (72%). These topics reflect foundational areas of training in pathogen genomics and are central to both research and public health applications. Other commonly reported topics included sample processing, library preparation, and sequencing workflows. In contrast, specialised areas such as biostatistics and systems administration were less frequently represented within the surveyed dataset. In this survey, “systems administration” referred broadly to managing computational environments, including Linux-based systems, high-performance computing (HPC) usage, workflow deployment, and cloud configuration. Respondents selecting “Other” topics reported additional areas such as antimicrobial resistance, use of the Terra platform for bioinformatics analysis, and infrastructure-focused capacity development, regulatory considerations for implementing genomics technologies, integration of “omics” data, and “train the trainer” approaches.

**Figure 3:**
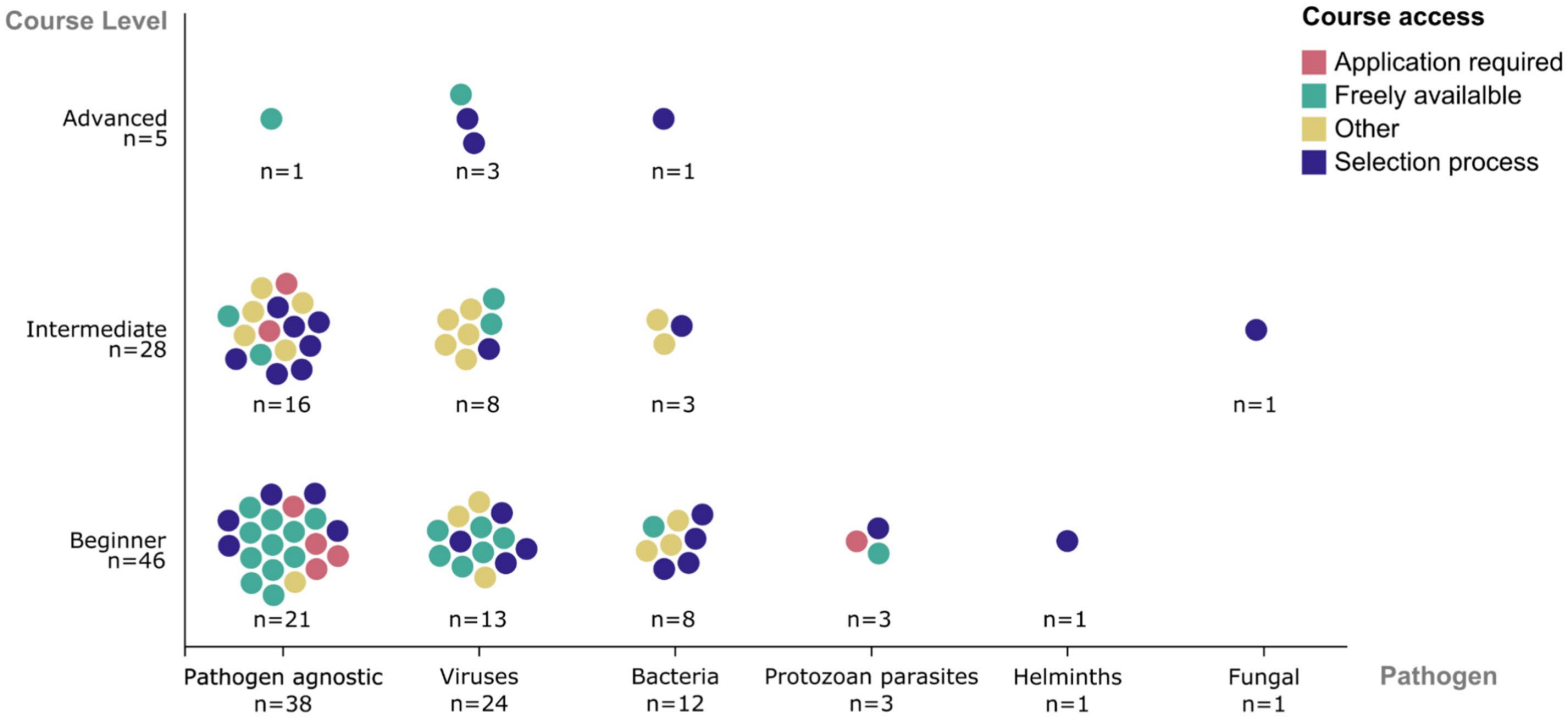
Course levels and participation models by pathogen focus. Distribution of surveyed training courses according to proficiency level, pathogen scope, and participation model. Each dot represents a single course and is color-coded by enrolment model: open access (teal), application required (red), selection process (purple), or other (yellow). “Other” includes paid courses or those delivered through funded partnerships with conditional access.

Among the 79 courses that reported a pathogen scope, 38 (48%) were not restricted to a single pathogen and instead focused on broadly applicable genomic methods relevant across multiple organisms (Figure 3). Among courses focused on specific pathogens, viral pathogens were the most commonly represented (*n* = 24; 30%), with over half of these related to *SARS-CoV-2* virus. Bacterial pathogens were covered in 12 courses (15%), while fungal pathogens, protozoan parasites, and helminths were each represented in fewer than 5 courses (6% combined).

Intended proficiency levels were also reported for the majority of courses. Beginner-level content predominated (*n* = 46; 58%), followed by intermediate-level offerings (*n* = 28; 35%). Advanced level courses were limited (*n* = 5; 6%). One metagenomics course excluded from the proficiency-level analysis was designed for participants at multiple skill levels and could not be classified within a single category.

### Application, selection, and attendance models

The surveyed courses employed a range of entry models (Figure 3). Twenty-five courses (31%) were fully open without application or payment requirements. A further 28 courses (35%) used a competitive but cost-free selection process to align participant backgrounds with course objectives. Seven courses (9%) required a simple application to confirm relevance or suitability, without additional selection criteria. The remaining 15 courses (18%) were categorised as “Other” including 10 requiring payment for attendance, and 5 delivered through funded partnerships or institutional arrangements.

When stratified by course level, entry models demonstrated increasing selectivity with course complexity. Among beginner-level courses, open participation was most common (41%), followed by selection-based access (33%). Intermediate-level courses more frequently required structured selection processes (36%), while advanced-level courses were primarily selective.

Participation models also varied by pathogen focus, although interpretation should account for the differing sample sizes across categories. Among courses not focused on a specific pathogen (*n* = 38), 14 (37%) were freely available, 6 (16%) required an application, 12 (32%) involved a competitive selection process, and 6 (16%) were delivered under the paid or funded arrangements. Among virus-focused courses (*n* = 24), 9 (38%) were freely available, 7 (29%) required a selection process, and 8 (33%) followed other enrolment models. Among bacteria-focused courses (*n* = 12), 6 (50%) required a selection process, 5 (42%) were delivered under paid or funded arrangements, and only 1 was freely available. Among courses addressing protozoan parasites, helminths, and fungal pathogens (*n* = 5 combined), only one course offered free access, with the remainder requiring either application or selection for attendance. Selection-based entry mechanisms were more frequently observed among intermediate and advanced courses, as well as among courses focused on specific pathogens.

### Assessment and accreditation

Knowledge or skills assessments were not included in 31 courses (38%). Learning activities such as group discussions or feedback sessions were reported in 8 courses (10%), although the extent to which these activities functioned as assessment mechanisms was not clearly specified. In contrast, formal structured assessments, including written examinations or multiple-choice quizzes, were reported in 6 courses (7%).

Certificates of completion were offered in 17 courses (21%), while 6 courses (7%) were linked to formal accreditation schemes, such as continuing professional development (CPD) credits awarded by recognised professional bodies. Twenty-seven courses (33%) did not provide any form of certification or accreditation. In several cases accreditation was reported as under consideration.

### Computing infrastructure

**Figure 4:**
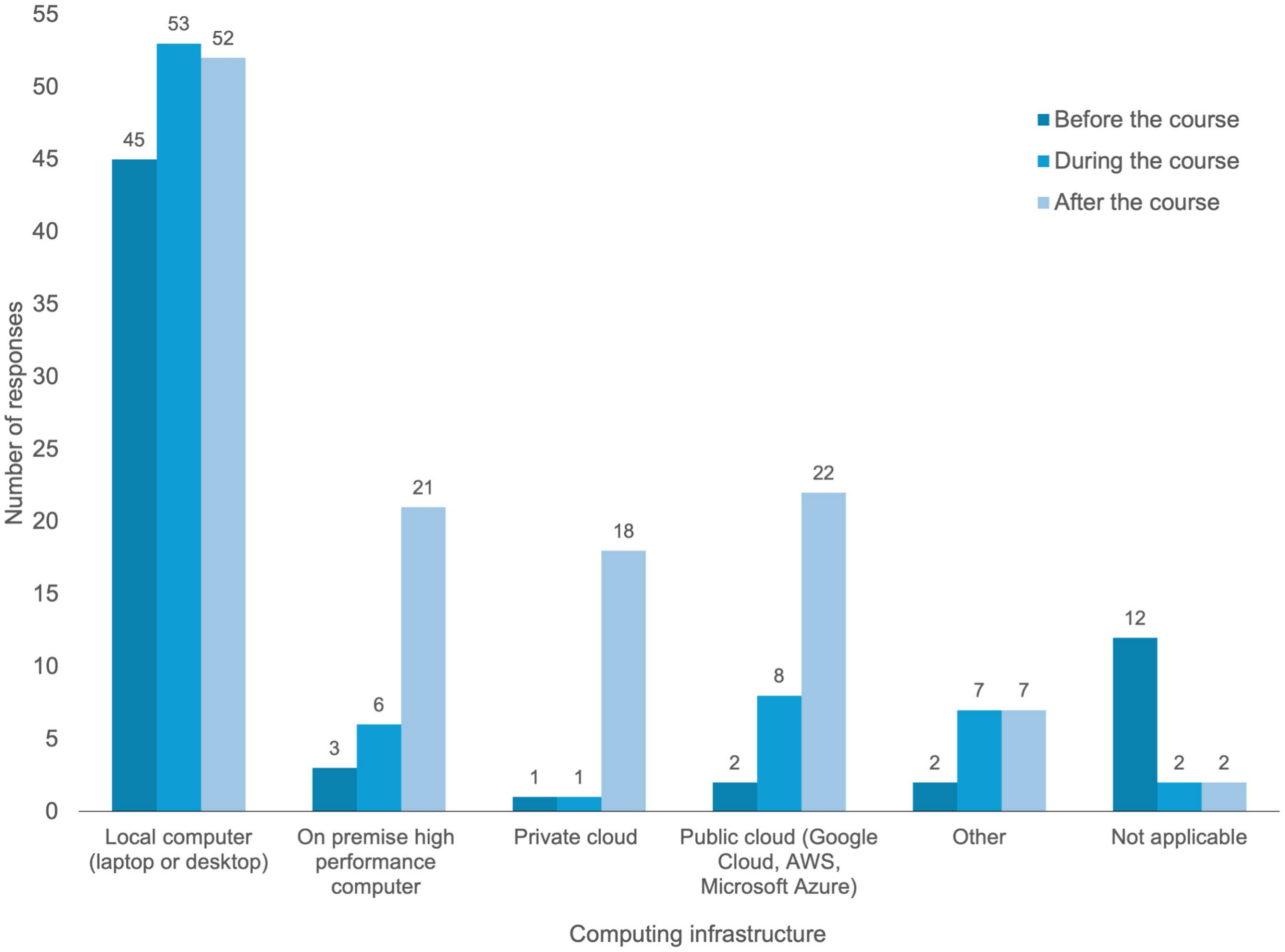
Computational infrastructure reported for pathogen genomics training delivery. The bar chart displays infrastructure types reported from a subset of course (*n* = 57). “Before” refers to infrastructure used in preparation for the course, “during” refers to infrastructure used during course delivery, and “after” refers to infrastructure reported as being used following course completion.

Computing infrastructure data were available for 57 of the 81 courses (70%). Percentages reported in this section are calculated using this subset. Local computing resources such as laptops or desktops were the most widely used resource across all phases of training (Figure 4). These were used in 45 courses (79%) before the training, increasing to 53 courses (93%) during training, and remaining high after training (91%). Use of on-premise high-performance computing (HPC) resources was reported in only 3 courses (5%) before the course, and in 6 courses (11%) during training increasing to 21 courses (37%) after training. Private cloud infrastructure was reported in 1 course (2%) before and during training, increasing to 18 courses (32%), after training. Public cloud platforms (such as Google Cloud, AWS, or Microsoft Azure) were reported in 2 courses (4%) before training, 8 courses (14%) during training, and 22 courses (39%) after training.

Other infrastructure types were reported in 2 courses (4%) before training, 7 courses (12%) during and after training. Twelve courses (21%) indicated that computing infrastructure was not required before training, decreasing to 2 courses (4%) during and after training. While most courses relied on local computing resources during delivery, a greater proportion reported use of institutional or cloud-based infrastructure following course completion.

## Discussion

This study provides a snapshot of pathogen genomics training initiatives identified through the PHA4GE Training & Workforce Development (TWD) Working Group and affiliated networks and should be interpreted as a mapped subset of training activity rather than a comprehensive global inventory. Survey responses described training courses associated with providers located across 17 countries. However, training providers located in the United Kingdom and the United States were disproportionately represented among the reported courses.

Although nearly one-third of face-to-face courses were specifically delivered outside the provider’s country and several initiatives were implemented across multiple regions over time, the institutional base of training provision was concentrated in a small number of higher-income countries. Similar disparities in genomics capacity have been documented elsewhere, reflecting broader inequities in investment, infrastructure, and training opportunities (8, 16). While locally and regionally tailored genomics programmes are essential, such initiatives remain unevenly distributed, and some regions continue to depend on externally based providers for specialised training. Strengthening sustainable, in-region training capacity remains critical to reducing structural imbalances in global genomics workforce development.

The predominance of short-format standalone courses reflects a training model that prioritises flexibility and responsiveness. Such formats can rapidly address emerging public health and emergency needs. However, when delivered independently of formal academic structures or professional accreditation systems, short courses may lack defined progression pathways and formal recognition structures, which could impact broader workforce development strategies. A recent study highlighted that structured training programmes and sustained collaboration with academic partners were identified as key enabling factors for strengthening interpretation capacity and long-term integration of genomic tools (17). University-affiliated programmes, fellowship-based models, and communities of practice offer more comprehensive learning experiences by integrating mentorship, expert guidance, and peer support, all elements that help cultivate deeper and more sustained expertise. Identifying how such models interact and scale alongside short-format courses could support more sustained capacity development across learner levels and professional contexts.

The survey findings demonstrate a strong representation of foundational concepts in pathogen genomic data analysis skills. Over 70% of courses covered bioinformatics pipelines, reflecting the central role these skills play in application of genomics in research, public health surveillance and clinical management. Wet-lab components such as sample processing, library preparation, and sequencing were also represented, indicating efforts to provide end-to-end understanding of genomic workflows. However, topics, such as biostatistics, systems administration, and infrastructure configuration were less frequently included, despite their importance in implementing comprehensive genomics workflows (18–21). For all courses, because topic inclusion was captured at a categorical level rather than reflecting depth or duration of instruction, these findings reflect relative representation of topics rather than the extent, intensity, or quality of their coverage.

Nearly half of surveyed courses were not restricted to a single pathogen and instead focused on broadly applicable genomic methods relevant across multiple organism types. Such foundational training supports transferable analytical skills that can be applied across research and public health contexts. Among courses centred on specific pathogens, viral pathogens were more frequently represented within the surveyed dataset. This likely reflects the heightened emphasis on SARS-CoV-2 during the study period, when genomic surveillance was central to rapid response efforts (17, 22–24).

While broadly applicable training remains essential for establishing core genomic competencies, there were fewer courses specifically addressing bacterial, fungal, or parasitic pathogens. As surveillance priorities evolve, particularly in relation to emerging infectious disease threats (25, 26), further examination of application-specific training coverage may be warranted. Modular training approaches that integrate foundational methods with targeted applications such as AMR genomics or metagenomic surveillance, may enhance preparedness and responsiveness across diverse public health settings (21, 27).

Regionally tailored programmes that integrate wet-lab sequencing, bioinformatics training, and train-the-trainer approaches illustrate how capacity development can be aligned with evolving pathogen surveillance priorities. Such models support sustainable workforce development in genomic data science for health by embedding mentorship, practical skills, and regional leadership development within training programmes (28–30). By empowering subject matter experts to lead training and shape contextually relevant priorities, these approaches move beyond one-off workshops toward sustained, locally anchored genomic capacity.

Most surveyed courses were classified as beginner level, with relatively few designated as advanced. This emphasis on foundational training reflects the prioritisation of baseline genomic competencies in many workforce development efforts. However, because courses were categorised into a single proficiency level and internal progression pathways were not explicitly captured, tiered or modular programme structures may not be fully visible in the current analysis. Competency-based frameworks and differentiated curricula such as those developed through Fogarty initiatives (31, 32), the NGS Academy (33), the Pathogen Genomics Competency Framework (32), and workshops such as VEME (34), demonstrate structured approaches to aligning learning objectives with diverse professional roles and experience levels.

Variation in entry models across course levels suggests increasing selectivity with course complexity. While open access was more common among beginner-level courses, intermediate and advanced offerings more frequently required selection or structured enrolment processes. These patterns may reflect efforts to align course complexity and resource requirements with participant background.

Although many courses reported serving participants from multiple countries, international reach does not necessarily equate to equitable accessibility. Flexible and multi-format approaches were common, with 40% of courses reporting more than one delivery mode. A substantial proportion of courses were delivered exclusively through remote, online or self-paced formats, including MOOC-based or pre-recorded models following the COVID-19 pandemic (7). While online delivery can broaden geographic reach and accommodate working professionals, participation depends on reliable internet connectivity, adequate computing resources, and language accessibility. At the same time, nearly half of courses reported a face-to-face component. In-person formats support hands-on laboratory instruction and peer interaction but may introduce barriers related to travel costs, visa requirements, institutional leave, and limited enrolment capacity (11). Delivery format therefore influences who is able to participate, depending on factors such as connectivity, travel requirements, and resource availability. Future work incorporating more detailed mapping of accessibility by country, delivery mode, and topic area could further clarify geographic participation patterns and structural barriers.

One of the persistent challenges in pathogen genomics training is the lack of mechanisms for monitoring course quality and assessing trainee competencies (35, 36). Fewer than 15% of courses incorporated structured evaluations or skills assessments. This may constrain opportunities for learners to formally demonstrate their competencies and for institutions to systematically evaluate training outcomes. While not all short-format programmes are designed to provide academic credit or formal certification, assessment plays an important role in verifying skills acquisition and supporting course quality improvement. This is particularly relevant in online and hybrid formats, where the absence of structured evaluation mechanisms may make it challenging to track learner progress or validate outcomes. In addition, many public health programmes may lack access to recognised accreditation mechanisms or ISO-aligned quality standards for pathogen genomics (1), contributing to variability in skill validation across contexts. Expanding the use of structured assessments and exploring context-appropriate accreditation pathways may strengthen both individual learning recognition and broader workforce capacity development.

Computational infrastructure is a central component of genomics training, underpinning participants’ ability to analyse, interpret, and manage data. The survey data indicate a progression in reported infrastructure use across the training timeline, with a shift from locally available computing resources toward more advanced platforms, such as high-performance computing (HPC) systems and cloud-based resources.

Local computers, particularly personal laptops and desktops, remained the most consistently used resource, reflecting their accessibility and practicality. This aligns with previous studies emphasising the importance of low-barrier computing environments for early-stage learners in genomics (27, 37). The increased reporting of HPC and cloud platforms post-training use suggests that some programmes successfully introduce participants to more advanced computing environments. However, the survey did not assess the depth of infrastructure instruction, the extent of hands-on engagement with these platforms, or participants’ long-term access to the diverse computing resources following course completion. In addition, sequencing platform specificity (e.g., Illumina, Oxford Nanopore, PacBio) was not captured in the survey. Because different platforms entail distinct computational workflows and cost considerations, incorporating platform-specific data in future work will provide clearer insight into how infrastructure training aligns with data generation laboratory practice.

The variability in reported platform use highlights the need to embed infrastructure literacy within training design. Beyond teaching analytical workflows, programmes should equip learners to navigate diverse computing environments, adopt reproducible practices and transition to infrastructure that supports independent application of skills (20, 38). Initiatives such as Data Carpentry’s Genomics workshop, ELIXIR Europe, and H3ABioNet exemplify how integrating training with practical access to computing infrastructure can support learners to move from analytic theory to scalable, infrastructure-enabled practice.

This analysis reinforces observations made by members of the PHA4GE TWD Working Group, regarding the difficulty of identifying pathogen genomics training programmes that are findable and accessible. Although training is delivered by diverse organizations, visibility remains variable, and coordination across providers is limited. The institutional diversity of training providers reflects a decentralised ecosystem. While this diversity enables context-sensitive programming, it may also limit coordination. Training efforts often operate in silos, with little shared visibility, leading to duplication and missed opportunities for collaboration. The curation of surveyed training information onto the NGS Academy platform (https://uct-cbio.github.io/ngs-academy/), represents one effort to improve discoverability of courses. Continued sharing of curricula, reusable resources, and quality indicators may further strengthen alignment across training initiatives.

**Figure 5:**
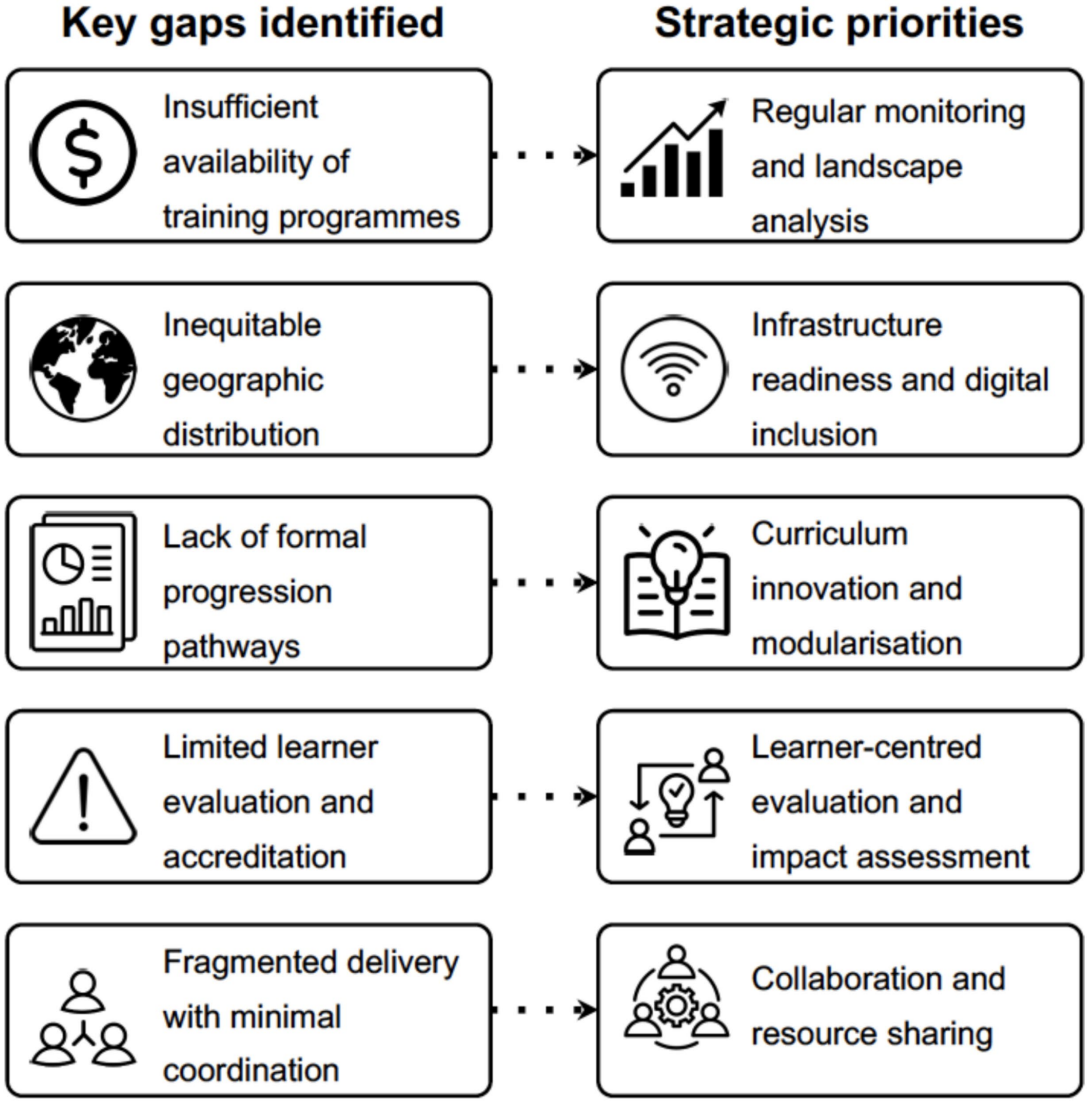
Alignment of structural challenges identified in the surveyed training initiatives with priority domains for pathogen genomics workforce development.

The findings of this landscape analysis highlight important patterns that align with the priority domains outlined by the PHA4GE TWD Working Group and illustrated in Figure 5, which focus on strengthening coordination, curriculum development, and integration of infrastructure capacity within pathogen genomics education. For example, limited visibility of training programmes aligns with the priority area of enhancing discoverability and coordination through continued landscape mapping and transparent reporting mechanisms. Similarly, variability in assessment and accreditation practices reflects the need to strengthen quality assurance and learner-centred evaluation approaches capable of supporting longer-term skill application and retention. The predominance of short-format and introductory courses within the surveyed dataset aligns with the priority of strengthening structured progression pathways. In addition, the variability observed in infrastructure access and coordination across providers corresponds with the strategic emphasis on infrastructure integration and ecosystem strengthening. Collaborative training ecosystems supported by shared platforms, reusable materials, and communities of practice may enhance coherence, reduce duplication, and improve alignment across initiatives.

Beyond documenting training programme characteristics, this landscape analysis provides a structured methodological approach that may inform future efforts to systematically examine distribution, accessibility, and equity in pathogen genomics workforce development. Applying similar mapping strategies across broader contexts could support improved coordination, resource sharing, and more inclusive capacity strengthening across regions.

### Study limitations

This study has several limitations that should be considered when interpreting the findings. First, survey dissemination occurred primarily through the PHA4GE Training & Workforce Development (TWD) Working Group and the professional contacts of its members. While this strategy enabled access to an engaged community of training providers active in pathogen surveillance across multiple regions, it limits generalisability beyond this defined dissemination frame and time period of data collection. Because the survey was circulated within this professional network, the resulting dataset reflects patterns of engagement and reported training activity rather than proportional geographic representation. The survey was administered in English only, which, although widely used in science and global health, may have influenced participation in regions where other languages predominate in professional or training contexts. When considered alongside the dissemination strategy, language accessibility may have shaped engagement patterns and contributed to lower representation from regions, including parts of Latin America and Asia.

Second, the study captures self-reported information from training providers and does not incorporate direct feedback from learners. Consequently, the analysis does not evaluate the depth of instructional content, learner experience, competency acquisition or long term workforce outcomes. Downstream impacts such as integration into surveillance systems, sustained infrastructure use, or policy influence were not assessed.

Third, topic coverage was captured at the level of categorical inclusion rather than instructional depth or duration. As such, representation of content areas should be interpreted as indicators of presence rather than intensity. Similarly, courses were classified into a single proficiency level, which may oversimplify modular or tiered programme structures. While infrastructure use was documented, the survey did not capture sequencing platform specificity (e.g., Illumina, Oxford Nanopore, PacBio), nor did it assess long-term access to institutional computing resources following training.

Finally, the dataset primarily reflects short-format and standalone, training courses. Embedded academic programmes, continuing professional development schemes, and long-term fellowships were not included. Findings should therefore be interpreted as describing one part of a broader and evolving training landscape.

Despite these limitations, the study provides empirical insight into structural characteristics of training provision within an active international consortium engaged in pathogen surveillance and offers a map of training across a defined professional network.

## Conclusion

This work provides a structured landscape analysis of pathogen genomics training initiatives identified within a defined network. The findings highlight observable structural patterns within the surveyed programmes, including geographic concentration of training providers, predominance of introductory formats and variability in assessment practices, and uneven integration of infrastructure-related competencies.

While the mapped training ecosystem demonstrates diversity in delivery models and thematic focus, visibility constraints, progression pathways and coordination across providers were variable. Improving discoverability, strengthening assessment mechanisms, clearer progression pathways, and enhanced infrastructure literacy may support more coherent workforce capacity development. The PHA4GE TWD Working Group contributes to efforts toward a more adaptable, inclusive and sustainable training ecosystem. By documenting training programme characteristics and aligning observed patterns with identified priority domains, this work contributes empirical evidence to inform future studies and capacity strengthening efforts in pathogen genomics training.

## Data Availability

The datasets presented in this study can be found in online repositories. The names of the repository/repositories and accession number(s) can be found in the article/Supplementary material.

## References

[1] Ballard SA Sherry NL Howden BP. Public health implementation of pathogen genomics: the role for accreditation and application of ISO standards. Microb Genom. (2023) 9:mgen001097. doi: 10.1099/mgen.0.0010972 37590046 PMC10483410

[2] Mwapagha LM. Why pathogen genomics is crucial in Africa’s public health. Afr J Lab Med. (2023) 12:21663. doi: 10.4102/ajlm.v12i1.21663 PMC10563014 37822518

[3] Onywera H Ondoa P Nfii F Ogwell A Kebede Y Christoffels A . Boosting pathogen genomics and bioinformatics workforce in Africa. Lancet Infect Dis. (2024) 24:e106–12. doi: 10.1016/S1473-3099(23)00394-8, 37778362

[4] Agboli E Bitew M Malaka CN Kallon TMPS Jalloh AMS Yankonde B . Building pathogen genomic sequencing capacity in Africa: centre for epidemic response and innovation fellowship. Trop Med Infect Dis. (2025) 10:90. doi: 10.3390/tropicalmed10040090 40278763 PMC12030795

[5] Webb JR Andersson P Sim E Zahedi A Donald A Hoang T . Implementing a national programme of pathogen genomics for public health: the Australian pathogen genomics program (AusPathoGen). Lancet Microbe. (2025) 6:1009696. doi: 10.1016/j.lanmic.2024.1009696 39389079

[6] Bishop M Miller E McPherson A Simpson S Sutherland S Seller A. Genomic education at scale: the benefits of massive open online courses for the healthcare workforce. Front Genet. (2019) 10:1094. doi: 10.3389/fgene.2019.01094, 31798624 PMC6863921

[7] Adkins J Carrell M. How COVID-19 changed the future of training. (2024). Available online at: https://www.calstatela.edu/business/businessforum/business-forum-vol-29-issue-2 (Accessed March 9, 2026).

[8] Nguinkal JA Zoclanclounon YAB Molina A Roba A Nyakio NM Lokamar PN . Assessment of the pathogen genomics landscape highlights disparities and challenges for effective AMR surveillance and outbreak response in the east African community. BMC Public Health. (2024) 24:1500. doi: 10.1186/s12889-024-18990-0, 38840103 PMC11151545

[9] Nadon C Croxen M Knox N Tanner J Zetner A Yoshida C . Public health genomics capacity assessment: readiness for large-scale pathogen genomic surveillance in Canada’s public health laboratories. BMC Public Health. (2022) 22:1817. doi: 10.1186/s12889-022-14210-9 36153510 PMC9508744

[10] Akande OW Carter LL Abubakar A Achilla R Barakat A Gumede N . Strengthening pathogen genomic surveillance for health emergencies: insights from the World Health Organization’s regional initiatives. Front Public Health. (2023) 11:11. doi: 10.3389/fpubh.2023.1146730, 37361158 PMC10289157

[11] Ras V Carvajal-López P Gopalasingam P Matimba A Chauke PA Mulder N . Challenges and considerations for delivering bioinformatics training in LMICs: perspectives from Pan-African and Latin American bioinformatics networks. Front Educ. (2021) 6:710971. doi: 10.3389/feduc.2021.710971

[12] Dunlop KLA Singh N Smit AK Morrow AL Steinberg J Cust AE . Building capacity for genomics in primary care: a scoping review of practitioner attitudes, education needs, and enablers. Front Med. (2025) 12:1577958. doi: 10.3389/fmed.2025.1577958, 40370713 PMC12076481

[13] World Health Organization. (2023). Global genomic surveillance strategy for pathogens with pandemic and epidemic potential 2022–2032: progress report on the first year of implementation. Available online at: https://www.who.int/publications/i/item/9789240084773 [Accessed June 23, 2025]

[14] Getchell M Wulandari S De Alwis R Agoramurthy S Khoo YK Mak TM . Pathogen genomic surveillance status among lower resource settings in Asia. Nat Microbiol. (2024) 9:2738–47. doi: 10.1038/s41564-024-01809-4, 39317773 PMC11445059

[15] Harris PA Taylor R Minor BL Elliott V Fernandez M O'Neal L . The REDCap consortium: building an international community of software platform partners. J Biomed Inform. (2019) 95:103208. doi: 10.1016/j.jbi.2019.103208, 31078660 PMC7254481

[16] Davedow T Carleton H Kubota K Palm D Schroeder M Gerner-Smidt P . PulseNet international survey on the implementation of whole genome sequencing in low and middle-income countries for foodborne disease surveillance. Foodborne Pathog Dis. (2022) 19:332–40. doi: 10.1089/fpd.2021.011 35325576 PMC10863729

[17] Bludau A Jack A Fischer N Dreesman J Drosten C Egelkamp R . Use of integrated genomic surveillance by local public health authorities: recommendations based on a mixed-methods study of current adoption, applications and success factors, Germany, 2023. Euro Surveill. (2025) 30:2400508. doi: 10.2807/1560-7917.ES.2025.30.13.2400508, 40183123 PMC11969963

[18] Atwood TK Bongcam-Rudloff E Brazas ME Corpas M Gaudet P Lewitter F . GOBLET: the global organisation for bioinformatics learning, education and training. PLoS Comput Biol. (2015) 11:1004143. doi: 10.1371/journal.pcbi.1004143, 25856076 PMC4391932

[19] Mulder NJ Adebiyi E Alami R Benkahla A Brandful J Doumbia S . H3ABioNet, a sustainable pan-African bioinformatics network for human heredity and health in Africa. Genome Res. (2016) 26:271–7. doi: 10.1101/gr.196295.115, 26627985 PMC4728379

[20] Baichoo S Souilmi Y Panji S Botha G Meintjes A Hazelhurst S . Developing reproducible bioinformatics analysis workflows for heterogeneous computing environments to support African genomics. BMC Bioinformatics. (2018) 19:457. doi: 10.1186/s12859-018-2446-1, 30486782 PMC6264621

[21] Onywera H Mulder N Kebede Y Tessema SK. How to sustain a public-health genomics and bioinformatics workforce in Africa. Nat Med. (2025) 31:2480–4. doi: 10.1038/s41591-025-03720-9, 40442296

[22] Poličar PG Špendl M Curk T Zupan B. Teaching bioinformatics through the analysis of SARS-CoV-2: project-based training for computer science students. Bioinformatics. (2024) 40:i20–9. doi: 10.1093/bioinformatics/btae208, 38940150 PMC11211835

[23] Lee KM Bosold A Alvarez C Rittenhouse D Felt-Lisk S Miller F . Surging the public health workforce: lessons learned from the COVID-19 response at state, tribal, local, and territorial public health agencies: ASPE Report. (2023). Available online at: https://www.ncbi.nlm.nih.gov/books/NBK603069/ (Accessed March 9, 2026). 38691039

[24] Weishaar H Pozo-Martin F Geurts B de Lopez Abechuco E Montt-Maray E Cristea F . Capacity-building during public health emergencies: perceived usefulness and cost savings of an online training on SARS-CoV-2 real-time polymerase chain reaction (qPCR) diagnostics in low and middle-income settings during the COVID-19 pandemic. Front Public Health. (2024) 12:1197729. doi: 10.3389/fpubh.2024.1197729, 38912269 PMC11192048

[25] Long C. Disease surveillance in the post-COVID-19 era. (2025). Available online at: https://www.e-ir.info/2025/05/14/disease-surveillance-in-the-post-covid-19-era/ [Accessed March 9, 2026]

[26] Struelens MJ Ludden C Werner G Sintchenko V Jokelainen P Ip M. Real-time genomic surveillance for enhanced control of infectious diseases and antimicrobial resistance. Front Sci. (2024) 2:1298248. doi: 10.3389/fsci.2024.1298248

[27] Founou LL Lawal OU Djiyou A Odih EE Amoako DG Fadanka S . Enable, empower, succeed: a bioinformatics workshop harnessing open web-based tools for surveillance of bacterial antimicrobial resistance. BMC Microbiol. (2025) 25:156. doi: 10.1186/s12866-025-03865-0, 40102762 PMC11921729

[28] Abrudan M Matimba A Nikolic D Hughes D Argimón S Kekre M . Train-the-trainer as an effective approach to building global networks of experts in genomic surveillance of antimicrobial resistance (AMR). Clin Infect Dis. (2021) 73:S283–9. doi: 10.1093/cid/ciab770, 34850831 PMC8634536

[29] COG-Train (2023). Establishing capacity for pathogen genomics 2023. Available online at: https://zenodo.org/records/11108958 (Accessed March 9, 2026).

[30] Lukhele ST Ras V Mulder N. Workforce development in genomic data science for health: a worldview. Annu Rev Genomics Hum Genet. (2025) 26:449–71. doi: 10.1146/annurev-genom-012224-122440, 40203237

[31] National Institutes of Health Fogarty International Center. Capacity-building in genomic surveillance and epidemiology. (2025). Available online at: https://www.fic.nih.gov/About/Staff/epidemiology-population-studies/Pages/Capacity-Building-in-Genomic-Surveillance-and-Epidemiology.aspx [Accessed August 18, 2025].

[32] National Institutes of Health Fogarty International Center. Multinational influenza seasonal mortality study (MISMS). (2022). Available online at: https://www.fic.nih.gov/About/Staff/multinational-influenza-seasonal-mortality-study-misms [Accessed August 18, 2025].

[33] NGS Academy for the Africa CDC. Pathogen surveillance training curricula. (2025). Available online at: https://uct-cbio.github.io/ngs-academy/courses/curricula/ [Accessed August 18, 2025].

[34] Virus Evolution and Molecular Epidemiology (VEME). Program. (2025). International Bioinformatics Workshop on Virus Evolution and Molecular Epidemiology. Available online at: https://zenodo.org/records/15720674 [Accessed August 18, 2025]. 10.1016/j.meegid.2013.08.023 24120112

[35] Campbell CE Nehm RH. A critical analysis of assessment quality in genomics and bioinformatics education research. LSE. (2013) 12:530–41. doi: 10.1187/cbe.12-06-0073, 24006400 PMC3763019

[36] Gurwitz KT Singh Gaur P Bellis LJ Larcombe L Alloza E Balint BL . A framework to assess the quality and impact of bioinformatics training across ELIXIR. PLoS Comput Biol. (2020) 16:e1007976. doi: 10.1371/journal.pcbi.1007976, 32702016 PMC7377377

[37] Kibet CK Entfellner JBD Jjingo D De Villiers EP De Villiers S Wambui K . Designing and delivering bioinformatics project-based learning in East Africa. BMC Bioinformatics. (2024) 25:150. doi: 10.1186/s12859-024-05680-2, 38616247 PMC11017571

[38] Cokelaer T Cohen-Boulakia S Lemoine F. Reprohackathons: promoting reproducibility in bioinformatics through training. Bioinformatics. (2023) 39:i11–20. doi: 10.1093/bioinformatics/btad227, 37387150 PMC10311340

[39] Nikolic D Abrudan M Kaldeli K Arruda LB Rey S Rodrigues C . Support Consistent, Competent Practice for Data Science in Pathogen Genomics: The Development of an Innovative Competency Framework The International Conference on Intelligent Systems for Molecular Biology (ISMB) 2024, Montreal: Zeonod. (2024).

