# Supplementary material for: Mapping pathogen genomics training provision: a structured analysis within a global consortium network
Source: Front Public Health. 2026 Apr 29;14:1768827. doi: 10.3389/fpubh.2026.1768827 (PMC13167935; doi:10.3389/fpubh.2026.1768827)
Supplement: Supplementary file 2 [file Data_Sheet_1.pdf]

A A A

PUBLIC HEALTH ALLIANCE FOR GENOMIC EPIDEMIOLOGY

H3ABioNet
Pan African Bioinformatics Network for H3Africa

Pathogen Genomics and Epidemiology Training Survey

The purpose of this survey is to assist in collating information and training materials for the various role players in pathogen surveillance and epidemiology. This forms part of our mission to contribute to the development of the global public health workforce.

We are gathering information about relevant courses that:

- are, or will be, available to participants from different regions and are accessible without registration costs **OR**
- have made course materials available for re-use

If you have more than one course, please complete this survey once for each course.

DETAILS OF RESPONDENT

Title:
* must provide value

First name(s):
* must provide value

Surname:
* must provide value

Email address:
* must provide value

ORGANISATION INFORMATION

Name of organisation:
* must provide value

Location of organisation (City, Country):
* must provide value

COURSE DETAILS

What is the title of the course?

* must provide value

Where can interested parties find out more about the course? Please provide a link.

* must provide value

In which context was the course developed and presented?

* must provide value

- Public health
- Research/academia
- Other

For which specific target audience was the course developed (e.g. bioinformaticians based at health institutes, experimental biologists at research institutions, trainers in bioinformatics settings)?

* must provide value

What are the primary focus areas of the course? Please select all options which are applicable.

* must provide value

- Epidemiology
- Pathogen genomics for clinicians
- Sample processing, DNA/RNA extraction
- Library prep
- Sequencing
- Bioinformatics
- Data analysis, data visualization, data management or a related topic (as would be applicable in the context of public health, pathogen genomics and epidemiology)
- Biostatistics
- Systems administration
- Other

Is the course focused on a specific pathogen or organism? If so, please elaborate.

Which sub-topics are covered by the course? Please provide a brief list or direct link to a course overview.

* must provide value

At what level is the course presented?

* must provide value

- ◯ Beginner
- ◯ Intermediate
- ◯ Advanced
- ◯ Other

Does the course include a formal assessment? If so, please describe the form of assessment which is used.

* must provide value

Does the course offer accreditation or certification? If so, please elaborate.

* must provide value

How is the course accessed?

* must provide value

- ◯ Course is freely available to anyone
- ◯ Course is available to anyone but an application is required
- ◯ Applicants undergo a selection process
- ◯ Other

How is the course presented? Please select all options which are applicable.

* must provide value

- ☐ Face-to-face sessions
- ☐ Live, remote sessions
- ☐ Pre-recorded materials
- ☐ Other

Is the course constantly available or is it presented at regular intervals?

Please comment on the duration and format of the course (e.g. 5-day workshop, hour-long seminar, hands-on tutorials over the course of two days).

* must provide value

Is the course content (in its current form) appropriate for participants from other regions?

* must provide value

- ◯ Yes
- ◯ No

Are subtitles or transcripts (either in English or in other languages) available to make content more accessible? If so, please elaborate.

* must provide value

Are the course materials available for re-use?

* must provide value

- ◯ Yes
- ◯ No
- ◯ Limited re-use

ADDITIONAL INFORMATION

Is there any other information you would like to add?

* must provide value

- ◯ Yes
- ◯ No

CONSENT

The data collected from this survey will be used to collate information and training materials in pathogen surveillance and epidemiology. Personal information provided such as email addresses will not be shared with third party members outside of the training program. All data collected will be stored in a clinical compliant REDCap database. Clicking on the Submit button will indicate you acknowledge this and are happy to submit your information.

Submit

Save & Return Later

Powered by REDCap
