# Supplementary material for: Mapping pathogen genomics training provision: a structured analysis within a global consortium network
Source: Front Public Health. 2026 Apr 29;14:1768827. doi: 10.3389/fpubh.2026.1768827 (PMC13167935; doi:10.3389/fpubh.2026.1768827)
Supplement: Supplementary file 3 [file Data_Sheet_2.pdf]

A A A

Follow-up: Pathogen Genomics and Epidemiology Training Survey

The purpose of this survey is to assist in collating information and training materials for the various role players in pathogen surveillance and epidemiology. This forms part of our mission to contribute to the development of the global public health workforce.

We are gathering information about relevant courses that:

- are, or will be, available to participants from different regions and are accessible without registration costs **OR**
- have made course materials available for re-use

If you have more than one course, please complete this survey once for each course.

DETAILS OF RESPONDENT

Title:
* must provide value

First name(s):
* must provide value

Surname:
* must provide value

Email address:
* must provide value

ORGANISATION INFORMATION

Name of organisation:
* must provide value

Location of organisation (City, Country):
* must provide value

COURSE DETAILS

What is the title of the course?

* must provide value

Prior to the course, participants should be familiar with the following compute infrastructure(s) (select all options which are applicable):

* must provide value

- ☐ Local computer (laptop or desktop)
- ☐ On premise high performance computer
- ☐ Private cloud
- ☐ Public cloud (Google Cloud, AWS, Microsoft Azure)
- ☐ Not applicable
- ☐ Other

During the course, participants will utilize the following compute infrastructure(s) (select all options which are applicable):

* must provide value

- ☐ Local computer (laptop or desktop)
- ☐ On premise high performance computer
- ☐ Private cloud
- ☐ Public cloud (Google Cloud, AWS, Microsoft Azure)
- ☐ Not applicable
- ☐ Other

After the course, participants will need access to the following compute infrastructure(s) to apply lessons learned during the training (select all options which are applicable):

* must provide value

- ☐ Local computer (laptop or desktop)
- ☐ On premise high performance computer
- ☐ Private cloud
- ☐ Public cloud (Google Cloud, AWS, Microsoft Azure)
- ☐ Not applicable
- ☐ Other

ADDITIONAL INFORMATION

Is there any other information you would like to add?

* must provide value

- ◯ Yes
- ◯ No

CONSENT

The data collected from this survey will be used to collate information and training materials in pathogen surveillance and epidemiology. Personal information provided such as email addresses will not be shared with third party members outside of the training program. All data collected will

be stored in a clinical compliant REDCap database. Clicking on the Submit button will indicate you acknowledge this and are happy to submit your information.

Submit

Save & Return Later

Powered by REDCap
